# Implementation and Evaluation of a School Nurse Toolkit to Reinforce Best Practices for Asthma Care in Schools

**DOI:** 10.5888/pcd21.240027

**Published:** 2024-08-22

**Authors:** Diane Wing, Evilia Jankowski, John Dowling, Tisa Vorce

**Affiliations:** 1 Wingspan Research Group, Grand Rapids, Michigan; 2 Michigan Department of Education, Lansing, Michigan; 3 Asthma Prevention and Control Program, Michigan Department of Health and Human Services, Lansing, Michigan

## Abstract

A toolkit, developed by a multidisciplinary team of national and statewide professionals, was promoted among school nurses in Michigan to support use of the standards of care for asthma in schools. We evaluated the effectiveness of the toolkit to assist school nurses in providing support for students with asthma. We used a multimethod approach to assess use of the toolkit, changes in nursing practices as a result of using the toolkit, and challenges encountered when implementing the standards for asthma care. During a 12-month period, from July 2022 through June 2023, increases in time on web page and monthly page views aligned with efforts to promote toolkit use. School nurses reported using the toolkit and implementing practice changes pertaining to training and education, ensuring proper use of and access to asthma medications, and advocating for self-carry of asthma medications. Challenges to implementing the standards of asthma care were time, parental engagement, institutional support, and identifying students with asthma. We found that our promotional efforts prompted school nurses to access the toolkit, which helped school nurses to effectuate practice changes to improve support for students with asthma in schools.

**Summary**

**What is already known on this topic?**

Resources reinforcing the standards of care for asthma can strengthen self-efficacy and use of asthma management practices among school nurses.

**What is added by this report?**

An asthma toolkit was promoted to school nurses in Michigan. School nurses viewed the toolkit and reported implementing changes to improve asthma management practices in their schools.

**What are the implications for public health practice?**

Reaching school nurses through promotional activities can encourage school nurses to apply the standards for care to support students with asthma in school.

## Introduction

The prevalence of current asthma in Michigan among children and adolescents aged 5 to 17 years is 8.8% (1). Of these children and adolescents, 35.3% missed 1 or more days of school due to asthma from 2017 to 2021 (2). Children and adolescents who are Black or in low-income households are disproportionately affected by asthma (2). From 2017 to 2021, 18.4% of Michigan children and adolescents aged 5 to 17 years with current asthma had an asthma-related visit to an emergency department or urgent care in the past 12 months; however, 30.9% of Black children and adolescents and 26.0% of children and adolescents in households with less than $50,000 in annual income had an asthma-related emergency department or urgent care visit in the past 12 months (2). Complex interactions among varying levels of social, structural, biological, and behavioral determinants contribute to asthma-related disparities (3).

In June 2022, the Michigan State Board of Education updated a model policy for supporting students with asthma that set forth recommendations for schools to establish asthma-friendly environments to improve students’ attendance and participation in activities and promote academic success and well-being (4). Building on the Whole School, Whole Community, Whole Child model (5), which provides conceptual support and practical guidance central to best practices, the policy designated school nurses as important members of a child’s support network to coordinate asthma management activities; integrate communication among students, caregivers, and health care providers; and ensure all school personnel have received appropriate training in asthma management and emergency response (4,5).

School nurses fill the gap between health care and education, provide both acute and chronic care, treat and assess behavioral health concerns, and connect students and families to community resources (6). Although school nurses are well positioned in their role to support students with asthma, inadequate time to devote to asthma management, due in part to competing student needs and multiple roles, impedes completion of these activities (7). Additionally, school nurses in Michigan practice in various models, and they may be responsible for covering more than 1 building or an entire school district. As such, a medically qualified person may not always be available to meet the emergent needs of students, and the oversight is shifted to school staff, teachers, and administrators (7).

Historically, Michigan has ranked lowest among states in its school nurse-to-student ratio (8). However, due in part to recent increases in school budgets (9), the number of employed school nurses has quadrupled since 2019, increasing from some 200 nurses to an estimated 800 nurses (Evilia Jankowski, MSA, BSN, NCSN, State School Nurse Consultant, Michigan Department of Education, October 5, 2023, email correspondence). Younger and less experienced school nurses have been reported to be less likely than older and more experienced school nurses to perform asthma management activities (10). Regardless of experience, however, asthma-focused education can strengthen self-confidence in asthma management among school nurses (10,11), and this self-confidence has been associated with increased performance of asthma management activities (10).

To support school nurses’ use of best practices for asthma management, a multidisciplinary team of national and statewide professionals convened to develop an asthma toolkit (12). The toolkit, *Supporting Students with Asthma at School: Standards of Care,* presents information for understanding asthma and applicable laws and details performance standards to support students with asthma (12). These standards include coordination of care, assembling health care plans, and training school personnel.

## Purpose and Objectives

The conceptualization of this evaluation was a collaborative effort between the Michigan Department of Education (MDE) and the Michigan Department of Health and Human Services (MDHHS) Asthma Prevention and Control Program. We designed the evaluation to assess the effectiveness of the toolkit to assist school nurses in providing support for students with asthma in schools. Our evaluation questions were 1) To what extent did school nurses use the toolkit? 2) What practice changes were implemented as a result of using the toolkit? and 3) What were the perceived challenges to implementing standards for asthma care in schools?

## Intervention Approach

On August 4, 2022, the toolkit was uploaded to the School Health Services page on the MDE website (12). The Michigan State School Nurse Consultant (SSNC) (E.J.) promoted the toolkit throughout the 2022–2023 school year. The SSNC introduced the toolkit at the Michigan School Nurse Summer Institute meeting in August 2022. The toolkit was promoted in the SSNC’s newsletter sent to school nurses in January 2023, during the SSNC’s monthly office hours in March 2023, and at the Michigan Association of School Nurses annual conference in May 2023.

## Evaluation Approach

The MDHHS institutional review board determined the evaluation to be exempt from full review and oversight. We used a multimethod approach, which consisted of collecting quantitative and qualitative data from several sources to facilitate a complete understanding of the extent to which the toolkit was used.

We used Google analytics to track the extent to which the home page of the toolkit was accessed from July 2022 through June 2023 on the MDE web page. The home page of the toolkit includes links to various components of the toolkit and a link to access and download a complete version of the toolkit document. We tracked time on page in seconds; the number of page views, defined as the number of times a web page was seen by all users; and the number of unique page views, defined as the average number of times a web page was seen by each user.

School nurses were invited to complete an online survey, administered by SurveyMonkey (www.surveymonkey.com), once in January 2023 and again in February 2023. We used the SSNC’s newsletter to invite the 800 school nurse subscribers to complete the survey. Two survey questions were used to assess toolkit use and practice changes made. The first question was, “Have you used the Asthma Toolkit to help guide you on asthma care and management for students in school?” Response options were yes and no. The second question was open-ended: “Based on your use of the Asthma Toolkit, have you made any process or practice changes in the way students with asthma are supported? If yes, briefly describe the changes made.”

In May 2023, the SSNC led a Kahoot! (www.kahoot.com) among school nurses attending the Michigan Association of School Nurses annual conference. A Kahoot! is a game-based platform used to collect real-time information from a group of people through a web browser on a mobile device. During the Kahoot!, 3 statements were used to collect information. The first was a true-or-false statement: “I have utilized the Asthma Toolkit on the MDE website.” The second was open-ended: “Share one practice change implemented since accessing the Asthma Toolkit.” The third was also open-ended: “What is the biggest challenge to implementing the standards of care for supporting students with asthma in school?” Responses to open-ended questions were limited to 250 characters.

We used Microsoft Excel to conduct a descriptive analysis of count data. We calculated frequencies and means for time on page, number of page views and unique page views, and we calculated frequencies for the number of respondents who used the toolkit. For qualitative analysis, we identified and developed themes on the basis of respondents’ comments, and we created and condensed categories on the basis of commonalities among the themes. We used Microsoft Excel to assign comments to a column and themes to a row to track when a theme was mentioned. One person (D.W.) coded the information and developed the coding scheme, which was reviewed and discussed with 3 team members (E.J., J.D., T.V.). Comments from the survey and Kahoot! were analyzed and reported separately.

## Results

Time on page totaled 6,124 seconds (1 hour, 42 minutes) and averaged 510 seconds (8 minutes, 30 seconds) per month. The least amount of time on page was 87 seconds in July 2022, the month before the toolkit was uploaded, and the greatest time on page was 1,049 seconds (17 minutes, 29 seconds) in March 2023. Page views totaled 819, averaged 68 per month, and ranged from 38 to 150. Unique page views totaled 648, averaged 54 per month, and ranged from 7 to 125. August and September had the greatest number of page views, and increases in page views generally aligned with efforts to promote toolkit use. The frequency of unique page views followed a similar pattern (Figure).

**Figure:**
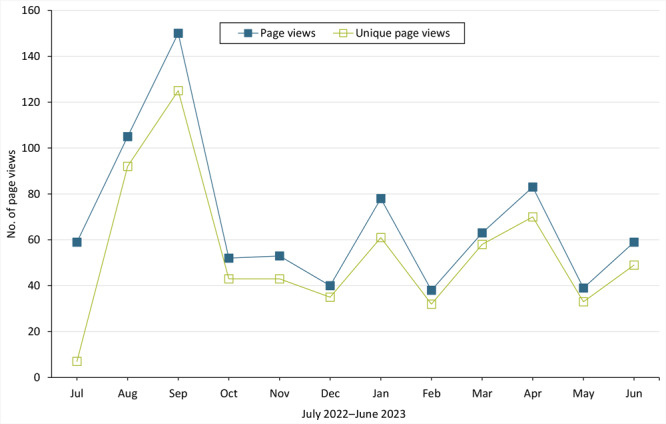
The number of page views (the number of times a web page was seen by all users) and unique page views (the average number of times a web page was seen by each user) of the Michigan Department of Education (MDE) web page linking to an asthma toolkit, July 2022 to June 2023, Michigan. The toolkit, *Supporting Students with Asthma at School: Standards of Care,* which presents information for understanding asthma and applicable laws and details performance standards to support students with asthma, was uploaded to the MDE website on August 1, 2022.

**Table:** 

Month	Page views	Unique page views
July	59	7
August	105	92
September	150	125
October	52	43
November	53	43
December	40	35
January	78	61
February	38	32
March	63	58
April	83	70
May	39	33
June	59	49

### Survey

Of the 800 school nurses who subscribed to the SSNC’s newsletter, 71 completed a survey (9% response). Of the 71 respondents, 42 (59%) indicated they used the toolkit, and of these, 11 (26%) provided information on practice changes made. We identified 2 themes based on comments: training and education and asthma action plans. Respondents reported using the information to educate administrators, staff, and families, using resources from the toolkit for training, and improving their own skills in recording asthma episodes. They also reported implementing the use of standardized asthma action plans and requesting and obtaining asthma action plans for each student with asthma.

### Kahoot!

Of the 176 meeting attendees, 140 participated in the Kahoot! (80% response). Of the 140 participants, 97 (69%) indicated that they used the toolkit, and of these, 73 (75%) provided information on practice changes as a result of toolkit use. Of these responses, 5 themes emerged: training and education (n = 25 respondents who made comments that pertained to a theme); asthma action plans or care plans (n = 19); use of asthma medications and spacers (n = 16); self-carry of asthma medications (n = 7); and other responses (n = 6).

School nurses reported using the guidance to train staff, some specifically referencing the tier-level training and infographic resources in the toolkit; educate staff, students, and families on asthma, such as proper inhaler use and asthma signs and symptoms; and improve their own knowledge, such as being more skilled in creating health care plans for students. Practice changes also included requesting asthma action plans to ensure all students with asthma had an asthma action plan on file, standardizing asthma action plans, or modifying care plans to be in accordance with performance standards. School nurses described changes made to ensure students had access to inhalers at school and during school-related events, as well as encouraging spacer use and following up with students after rescue inhaler use. School nurses reported advocating for self-carrying medications in their school, ensuring staff were aware of students who self-carry medications, or sharing asthma action plans with teachers to allow students to safely self-carry medications; 1 respondent described asking students who self-carry medications to demonstrate proper inhaler use. Other practice changes related to completing asthma assessments, providing support for asthma trigger reduction, collecting data, checking oxygen saturation regularly, and encouraging medical provider visits.

When school nurses were asked to describe their biggest challenge to implementing the standards for asthma care in school, of the 97 participants who used the toolkit, 57 (59%) provided a brief description of challenges. Time (eg, being busy), parents (eg, parental involvement, communication, support, obtaining asthma action plans), support (eg, staff compliance, administrator buy-in, physician response, being understaffed), and being able to identify students with asthma were reported challenges. Of the 43 respondents who did not use the toolkit, 22 (51%) briefly described challenges as time, parents (eg, parental involvement, communication, cooperation, support), and communication and collaboration.

## Implications for Public Health

We aimed to identify the types of practice changes made as a result of toolkit use. Our findings suggest the toolkit reinforced practices for training of staff on the tier levels for asthma management and emergency response training and with educating staff, students, and families on asthma and proper use of asthma medications. School nurses also reported standardizing asthma action plans, increasing the number of students’ asthma action plans on file, and advocating for self-carry of asthma medications in schools. Some school nurses improved their own skills with recording asthma episodes and developing care plans. We gained a limited understanding of challenges encountered by school nurses when implementing the standards of care for asthma; these challenges were noted in previous research (7,13). Introducing a model for school nurse–led management can help schools meet the health needs of students with asthma, especially those with multiple and complex barriers to health and academic success, and provide professional support for school nurses to moderate challenges (14).

Evaluation findings confirmed that our promotional efforts prompted school nurses to access the toolkit. School nurses reported using the toolkit, and the uptick in web page visits and time on page generally corresponded with promotional activities. Replication of this approach could be used to promote additional toolkits developed for school nurses to support students with other health conditions.

Our study has several limitations. Findings are not generalizable to all school nurses due to low response rates. Additionally, survey and Kahoot! respondents were reached through the SSNC’s newsletter and a Michigan Association of School Nurses conference and may not reflect the entire population of school nurses. A school nurse could have participated in the survey and Kahoot! and may be represented more than once. We did not collect information on demographic characteristics of school nurses in Michigan. Practice changes were based on self-report and could not be externally validated, and our understanding of aspects of the toolkit that school nurses deemed most useful was limited. Additionally, the scope of the evaluation did not examine whether practice changes led to improved asthma outcomes for students.
